# Pharmacological Studies Pertaining to Smooth Muscle Relaxant, Platelet Aggregation Inhibitory and Hypotensive Effects of* Ailanthus altissima*

**DOI:** 10.1155/2019/1871696

**Published:** 2019-03-03

**Authors:** Hafiz Muhammad Abdur Rahman, Muhammad Fawad Rasool, Imran Imran

**Affiliations:** ^1^Department of Pharmacology, Faculty of Pharmacy, Bahauddin Zakariya University, 60800 Multan, Pakistan; ^2^Department of Pharmacy Practice, Faculty of Pharmacy, Bahauddin Zakariya University, 60800 Multan, Pakistan

## Abstract

**Objective:**

This in vitro and in vivo study was conducted to rationalize some of traditional medicinal uses of* Ailanthus altissima* in gastrointestinal, respiratory, and cardiovascular systems.

**Materials:**

Crude extract of* Ailanthus altissima* (Aa.Cr) and its fractions were prepared and utilized in in vitro and in vivo studies. For in vitro studies, Aa.Cr was investigated on isolated rabbit jejunum, isolated tracheal strip, and isolated aorta of rat suspended in tissue organ bath. Platelet rich and platelet poor plasma were used to study platelet aggregation inhibitory activity. In vivo antidiarrheal effect of Aa.Cr was investigated on balb/c mice pretreated with castor oil to induce diarrhea and SD rats were used to study hypotensive activity.

**Results:**

Concentration dependent spasmolytic effects of Aa.Cr and its DCM fraction (Aa.DCM) were observed on spontaneous and spasmogen induced contractions in jejunum isolated from rabbit, but effect against high potassium (high-K^+^) induced contractions was more potent. Moreover Aa.Cr showed parallel shifting of calcium response curve to the right side. While its aqueous fraction (Aa.aq) caused spasmogenesis of isolated rabbit jejunum, this effect was blocked partially with prior administration of atropine (1*μ*M). Concentration dependent protection against castor oil induced diarrhea was also observed. Relaxant effect was observed by the application of Aa.Cr and Aa.DCM against high-K^+^ and carbachol (CCh) induced contractions in tracheal strips isolated from SD rats, while Aa.Aq caused partial relaxation of high-K^+^ induced contractions, but no effect was observed against CCh induced contractions. Relaxation of rat aorta by the application of Aa.Cr and its fractions was also observed. Inhibition of force of contraction in rabbit atrium was also observed. Inhibition of platelet aggregation was observed against epinephrine and ADP induced aggregation.

**Conclusion:**

Keeping in view the observed results, it is concluded that smooth muscle relaxant, platelet aggregation inhibitory and hypotensive effect may be due to the blockage of calcium channels.

## 1. Introduction

*Ailanthus altissima* (Synonyms:* Ailanthus cacodendron *(Ehrh.) and* Ailanthus glandulosa *Desf.) belongs to Simaroubaceae family and is commonly called tree of heaven, smoke tree, God's tree, Chinese sumac, and sumac tree [1]. It is native to China, Indian occupied Kashmir, Vietnam, Taiwan, and Japan [2, 3]. This plant has peanut or cashew like fragrance [4, 5]. It has strong ornamental acceptance and due to this reason has become naturalized in many countries even in Pakistan [6].

*A. altissima* is widely used throughout Asia for the treatment of several disorders in traditional medicine. Its dried bark is listed as an official drug in modern Chinese materia medica as “chun bai pi”. This plant is used to treat gastrointestinal ailments like diarrhea, dysentery [7, 8], vermicide, and hemorrhage of intestine [9]. It is also used to cure leucorrhea, gonorrhea, cough, and gastric and intestinal complaints [10]. The bark is used to treat anemia, hemorrhage, diarrhea, and spermatorrhea and is considered as useful to treat epilepsy, diarrhea, asthma, and cardiac problems. In Chinese system of medicines, it is used to treat colds and gastric problems [11]. The leaves of* A. altissima* are used for the treatment of scabies and seborrhea [12]. Its bark is astringent, antispasmodic, anthelmintic, and parasiticidal [7, 13]. It possesses phosphodiesterase inhibitory [14], angiotensin converting enzyme inhibitory, analgesic, anti-inflammatory, antipyretic, antioxidant, and antiasthmatic activities [15]. Previous reported literature indicates that it possess pain reducing, ulcer healing, antipyretic [10], anticancer [16], COX inhibitory, and antioxidant potentials [17]. Also it has been reported that* A. altissima* possess antimicrobial and antifungal activities [18].

It has been reported that* A. altissima* contains hundreds of chemical constituents. A number of alkaloids with basic structure of *β*-carboline, canthine-6-one and canthine-6-one-3 N-oxide and indole have been reported [19, 20]. Presence of quercetin and isoquercetin is in leaves, ceryl alcohol in bark, 2,6-dimethoxy-p-benzoquinone, ailanthone, ailanthinone, chaparrinone, amarolide, and acetylamarolide in the wood of* Ailanthus altissima *[21, 22], while 1-acetyl-4-methoxy-*β*-carboline (I), canthin-6-one (VIIIa), 1-methoxycanthin-6-one (VIIIb), canthin-6-one-3N-oxide (IXa), 1-(2'-hydroxyethyl)-4-methoxy-*β*-carboline (IIa), 1-(1', 2'-dihydroxyethyl)-4-methoxy-*β*-carboline (IIIa), and 1-methoxycanthin-6-one-3N-oxide (IXb) have been isolated from its root [22, 23]. It has been reported that* A. altissima* possess (E,E)-2,4-decadienal, (E)-2-undecenal, (E)-2-decenal, hexanal, nonanal and furfural which have nematicidal activity [24]. Presence of flavonoids in this plant has also been reported [2].

## 2. Material and Experimental Protocols

### 2.1. Plant Collection and Extraction

Bark of* A. altissima *was collected from the botanical garden of Bahauddin Zakariya University, Mulan, Pakistan, authenticated and identified by a taxonomist (voucher no: R.R. Stewart 607). After shed drying, the bark material was grounded into coarse powder. One kilogram grounded bark was macerated in 2.5 liter of hydroalcoholic solution (30% water and 70% methanol). The macerate was filtrated by using muslin cloth and Whatman filter paper. The filtrate was dried on a rotary evaporator at controlled pressure and temperature (37°C) [25, 26], and a semisolid or honey like material was obtained. For the purpose of fractionation, 50g dried extract of* Ailanthus altissima* (Aa.Cr) was dissolved in 100 mL of distilled water and equivalent volume of dichloromethane was added to it. This solution was shifted to separating funnel and gently shaken and then kept on a stand. Two separate layers of solution were formed; both layers were separated and dried.

### 2.2. Drugs and Chemicals

High quality chemicals and drugs were used in this study and were purchased from reliable resources, i.e., Sigma Aldrich (merged with Merck Ag, Germany), Merck (Germany), and Alfa Acer (USA). Acetylcholine (Ach), carbachol, atropine sulphate, loperamide, verapamil hydrochloride, and potassium chloride were used as standard drugs for in vivo and ex vivo studies. Sodium chloride (NaCl), calcium chloride (CaCl_2)_, sodium dihydrogen phosphate (NaH_2_PO_4)_, potassium dihydrogen phosphate (KH_2_PO_4_), magnesium chloride (MgCl_2)_, magnesium sulphate (MgSO_4)_, potassium chloride (KCl), glucose (C_6_H_12_O_6_), and sodium citrate were used in the preparation of physiological salt solution. Before the execution of experiments, all solutions were freshly prepared in double deionized water.

### 2.3. Animals

Locally bred rabbits (1.2–1.8 kg), Sprague Dawley rats (150–300g), and BALB/c mice (20–35g) either males or females were used in this study. These animals were placed in the animal house of Pharmacology Department, Faculty of Pharmacy, Bahauddin Zakariya University, Multan. Temperature of the animal house was maintained at (23-25°C), humidity (50-55%) and 12 h light and dark cycle. Standard diet was provided to animals with full access to water. 16-20 hours before the start of experiments freedom for food was banned but* ad libitum* water. The rules set up by the Institute of Laboratory Animal Resources, Commission on Life Sciences, National Research Council (1996) were followed in the execution of experiments. Care was exercised for the minimal number of animal usage defined by the principle of 3Rs, i.e., Replacement, Reduction, and Refinement. 3Rs principle was applied for the minimal number of animal usage. The protocols of this study were approved by the departmental ethical committee vide No.09/PEC/2015.

### 2.4. Preliminary Phytochemical Analysis

To analyze the presence of bioactive molecules like alkaloids, saponins, flavonoids, anthraquinones, tannins, and glycosides in plant extract, the following tests were performed [27–29].

#### 2.4.1. Alkaloids

Plant extract was dissolved in distilled water. This solution was shifted into four different test tubes. Few drops of reagents, i.e., Mayer's reagent, Hager's reagent, Wagner's reagent, and Dragendorff's reagents, were added separately to these test tubes. Formation of precipitates indicated the presence of alkaloids.

#### 2.4.2. Flavonoids

One gram of plant extract was dissolved in H_2_SO_4_ and boiled then filtered. 1% NH_3_ solution was added to the filtrate drop wise and observed for any change in color. Appearance of yellow color was considered as the presence of flavonoids.

#### 2.4.3. Anthraquinones

1 gram of plant extract was boiled in H_2_SO_4_; this solution was treated with 10 mL of chloroform. By the addition of chloroform solution split into two layers, both layers were separated out and the upper layer was treated with dil. Ammonia solution. Observation of violet color was considered as indication for the presence of anthraquinone.

#### 2.4.4. Saponins

1 gram of test material was dissolved in 10 mL of distilled water by vigorous shaking until the formation of froth. Then few drops of olive oil were added to it. Formation of emulsion or froth formation indicated the presence of saponins.

#### 2.4.5. Tannins

For the identification of presence of tannins in plant extract, aqueous solution of plant extract was treated with drop wise addition of 1%FeCl3 solution. Appearance of bluish-black or brownish-green color indicated tannins presence.

#### 2.4.6. Reducing Sugar Test

Mixture of aqueous solution of plant extract and Fehling's solution was heated. Formation of brick red color precipitates indicates reducing sugars.

#### 2.4.7. Deoxy-Sugar in Glycosides

For the detection of deoxy sugars in glycosides, Keller-Killiani test was adopted. In this test plant extract was dissolved in chloroform. This solution was treated with H_2_SO_4_ carefully along the wall of test tube. If deoxy sugars are present in the sample, then brown color will appear at the interface.

### 2.5. Ex Vivo Experiments on Isolated Tissues

#### 2.5.1. Experiments for Spasmolytic Activity on Jejunum Preparation

Rabbit jejunum was used to study the possible spasmolytic activity of Aa.Cr and its fractions. After scarifying the rabbit, jejunum was removed and small pieces about 2–3cm in length were made. The jejunum pieces were suspended in 15 mL tissue bath filled with normal Tyrode's solution. The temperature of the bath was kept constant at 37°C with continuous supply of carbogen and a tension of 1g was applied to the tissue. The tissue was kept free to equilibrate for a period of 30–45 minutes and during this period no drug was applied, but it was repeatedly washed with fresh Tyrode's solution. Then the tissue was stabilized by repeated exposure to acetylcholine (0.3*μ*M) after each 3-minutes interval. Spasmolytic action of plant material was investigated by cumulative administration of test drug. This effect was measured as percentage of jejunum spontaneous contractions obtained immediately before the application of 1^st^ dose of test materials. Calcium channel blocker activity was studied by the application of Aa.Cr to the jejunal strip preexposed to 80 mmol/L K^+^ [26, 30, 31]. To confirm that the spasmolytic activity of the* A. altissima* is due to the blockage of calcium channel or any other mechanism involved, concentration response curves of calcium (CRCs) were constructed. For this purpose, the tissue was stabilized in calcium free and K^+^ rich Tyrode's solution. After the stabilization and incubation, the tissue was treated with Aa.Cr and left free for a period of 45 minutes. Then calcium was administered in a cumulative manner and the contractile response was observed [32].

#### 2.5.2. Study of the Relaxant Effect on Isolated Rat Tracheal Tissue

To study the relaxant effect of the test drug rat tracheal tissues were used. 24-hour fasted rat was killed by a sharp blow on its head and the trachea was carefully separated. After removing the adhesive tissues, the trachea was cut open ventrally. Small tracheal strips with a width of 3-4 mm having minimum 2-3 cartilages were formed. These strips were individually hung in a tissue organ bath containing Krebs's solution at 37°C with constant carbogen perfusion. 1g tension was applied to tissue till the end of experiment. After hanging, the tissue was allowed to equilibrate up to 45 minutes prior to the application of any drug. After equilibration, the tissue was exposed to 80 mmol of potassium (high-K^+^) or carbachol (1*μ*M) to induce contractions in it. Then relaxant effect of plant extracts was observed on these precontracted tracheal tissues via isometric transducers (MLT 0201, Panlab, Spain) connected to PowerLab data acquisition system [25, 30].

#### 2.5.3. Vasodilation Study on Isolated Rat Aorta

Vasodilation potential of plant extract and its fractions was evaluated by using isolated rat aorta with adaptation of the reported procedure [18–20, 22]. SD rat was sacrificed by a sharp blow on its head and descending thoracic aorta was dissected out and pieces of 2–3mm were formed. Each isolated rat aortic tissue segment was suspended in tissue organ bath filled with Kreb's solution. Temperature of tissue organ bath was fixed at 37°C with continuous supply of carbogen. Then, 2g preload tension was applied to the tissue and left it to equilibrate for a period of 1h. Tissue was repeatedly exposed to phenylephrine (1*μ*M) or high potassium (K^+^-80 mM) for the purpose of stabilization. The vasodilator effect of plant material was studied by cumulative addition of plant extract to precontracted aortic rings. Changes in the tension of precontracted aortic rings were measured via isometric transducer MLT 0201 (Panlab, Spain) which was in connection with PowerLab data acquisition system (ADInstruments, Australia) coupled with LabChart software. Vasodilation effect of the test material was calculated as %age relaxant effect by considering the base line response of the tissue as 100% relaxant effect and PE and high-K^+^ contractions as 100% contractile effect.

#### 2.5.4. Study of Ionotropic and Chronotropic Effect on Isolated Rabbit Atria

Right atrium was isolated carefully from heart of rabbit and hung in tissue organ bath having Kreb's solution with the continuous supply of carbogen. Ionotropic and chronotropic effect of atria were recorded under 1.0 g pressure by isometric transducer associated with PowerLab. Atrial tissues were permitted to equilibrate for a time of 30 min with persistent buffer change at regular intervals preceding administration of any drug [21]. After stabilization of the tissue, it was exposed to isoprenaline (1*μ*M) and acetylcholine (1*μ*M) to check the responsiveness of the tissue. Then test materials were administered cumulatively and the responses were noted on rate and force of contractions. Negative inotropic and chronotropic effects of the test drugs were calculated as %age of the baseline response of isolated atrium.

#### 2.5.5. Study of Platelet Aggregation Inhibitory Activity

Platelet aggregation inhibitory effect of the plant extract was determined by using the method as described by Imran et al. (2015). Blood samples were collected from the healthy human volunteers with age range of 24±4 who have not taken any medicine at least one week prior to experimentation. Sodium citrate (3.8% w/v) solution as anticoagulant was mixed with blood in a ratio of 9:1 (blood: sodium citrate solution). Blood sample was subjected to centrifugation at a speed of 1500 rpm for 15 minutes. Supernatant was separated out as platelet rich plasma (PRP). Remaining blood sample was further centrifuged at a speed of 4,000 rpm for a period of 20 minutes to get platelet poor plasma (PPP). Antiplatelet activity of the test drugs was determined by using chronolog 490-2D aggregometer (Chrono-log Corporation, Haverton, PA 19083, USA) which works on the principal of light transmission aggregometry (LTA). After maintaining the temperature of the machine, 0.23 mL of PRP was taken in a siliconized microcuvette with spacer at the bottom and placed in PRP chamber. Final volume of the cuvette was made up to 0.25 ml by the addition 10 *μ*L of standard aggregating agent ADP (5*μ*M) or phenylephrine (40 *μ*M) and 10*μ*L of extract dissolved in normal saline. Similarly, 450 *μ*L of PPP was taken in a cuvette and placed in PPP chamber as a reference. PRP was first incubated with 10*μ*L of test material for a period of five minutes then 10*μ*L of ADP or PE was added to it to make up the volume 0.25 mL, placed in PRP chamber, and response was observed. Results were calculated as percentage inhibition with respect to time.

### 2.6. In Vivo Experiments

#### 2.6.1. In Vivo Antidiarrheal Activity

BALB/c mice were segregated into six groups randomly having six animals in each. These mice were placed in individual cages which were lined with blotting paper. On the day of experiment, all animals were orally exposed to castor oil (10 mL/kg) for the induction of diarrhea. One hour after castor oil treatment, Group-I animals (negative control) were treated with 0.9% sodium chloride solution (10 mL/kg) while group-II (positive control) was orally given loperamide (15 mg/kg). While group-III was orally treated with Aa.Cr (100 mg/kg), group-IV with Aa.Cr (200 mg/kg), group-V with Aa.Cr (300 mg/kg), group-VI was treated with Aa.Cr (500 mg/kg). After treatment all animals were observed up to 6 h and total number of feces was counted individually for each animal [33].

#### 2.6.2. Invasive Hypotensive Activity

Hypotensive activity of plant extracts was studied on SD rats by the previously described method [21, 24]. Ketamine (50–80 mg/kg) and diazepam (5mg/kg) were used intraperitoneally to anesthetize the rats. The anesthetized rat was placed in a supine position on dissecting board. Temperature of the rat was kept constant by putting it on isothermic warming cushion at 37°C. Trachea, jugular vein, and carotid artery were uncovered by making a small incision and cannulated. Spontaneous breathing was maintained by cannulation of trachea with 18 gauge polyethylene tube (PE-20). The jugular vein was cannulated to administer drug/s with PE-50. Cannulation was performed in carotid artery with similar tubing filled with heparin solution (60 IU/mL) and attached with disposable BP transducer (MLT0699, ADInstruments, Sydney, Australia), further connected to PowerLab data acquisition system coupled with LabChart software for the measurement of blood pressure. The transducer was calibrated prior to experiment with the help of mercury sphygmomanometer. Surface exposed for cannulation was kept covered with filter paper moistened with normal saline. The rat was administered 0.1 mL of heparin to prevent clotting of blood. Standard medications were administered in 0.1mL followed by 0.1mL of 0.9% normal saline flush through the jugular vein. To check the animal behavior towards hypertensive and hypotensive drugs acetylcholine (1*μ*g/kg) and epinephrine (1*μ*g/kg) were administered intravenously to every animal before the administration of any drug. After attaining the equilibrium, 0.1 mL of the extract or drug was administered intravenously followed by normal saline (0.1 mL) flush [24]. Changes in systolic blood pressure (SBP), diastolic blood pressure (DBP), and mean arterial pressure (MAP) were calculated and compared with the response of verapamil used as standard hypotensive drug.

### 2.7. Acute Toxicity Assay

For acute toxicity assay, three groups of mice, five mice in each group, were selected. 1^st^ group was given Aa.Cr 1 g/kg orally while the 2^nd^ and 3^rd^ groups received Aa.Cr 2 g/kg and 3 g/kg in a volume of 10 mL/kg, respectively. The animals were observed for 24 h for behavioral changes and mortality but with full access to water and food [25, 31].

### 2.8. Statistical Analysis of Data

For isolated tissues, the data were represented as mean ± SEM (*n* = number of individual experiments) and median effective concentrations (EC_50_) with 95% confidence intervals. The concentration response curves were analyzed by nonlinear regression. While Two-Way ANOVA followed by Tukey's multiple comparison test was used to determine the significant difference in various concentration, Student t-test was utilized to analyze hypotensive effect. One-Way ANOVA followed by Dunnett's multiple comparison test with defined control group as a reference was used for the analysis of antidiarrheal and antiaggregatory activities. In all cases,* P* values <0.05 were considered to be statistically significant. All statistical tests were performed by using GrphPad prism software (V 6.01).

## 3. Results

### 3.1. Preliminary Phytochemical Reports

Phytochemical evaluation of Aa.Cr showed that alkaloids, saponins, tannins, glycosides, flavonoids, and triterpenoids were present in it.

### 3.2. In Vitro Activities

#### 3.2.1. Spasmolytic Effect on Isolated Jejunum of Rabbit

**Figure 1:**
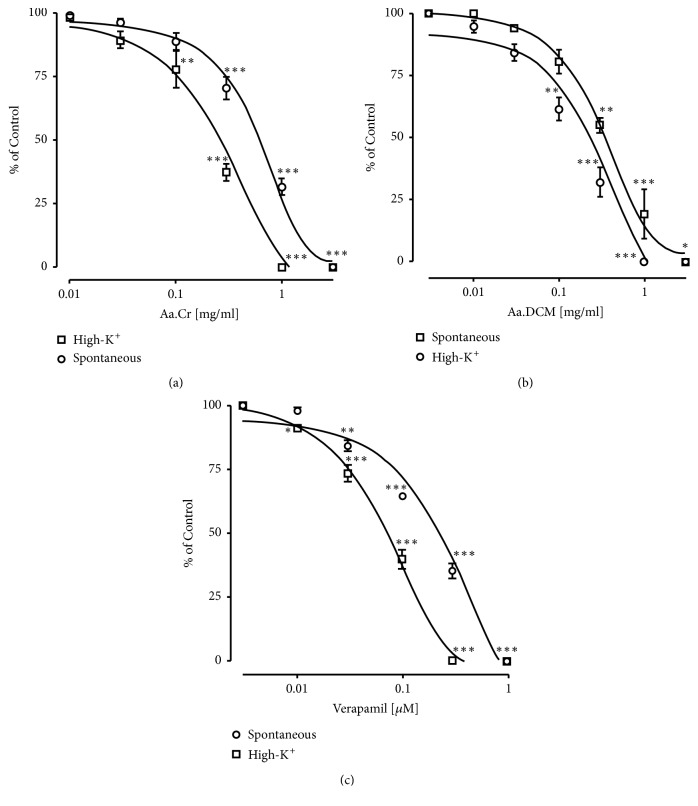
Concentration response curves showing concentration dependent spasmolytic effect of (a) Aa.Cr, (b) Aa.DCM, and (c). Verapamil on spontaneous and high-K^+^ induced contractions in isolated rabbit jejunum. Data is represented as mean ± SEM of 6-8 individual experiments and statistically analyzed with the implication of Two-Way ANOVA followed by Tukey's multiple comparison test.  ^*∗*^ *P*<0.05,  ^*∗∗*^ *P*<0.01,  ^*∗∗∗*^ *P*<0.001 shows a comparison of concentration dependent effects on the basis of individual doses (specified effect compared with the effect of preceding dose).

**Figure 2:**
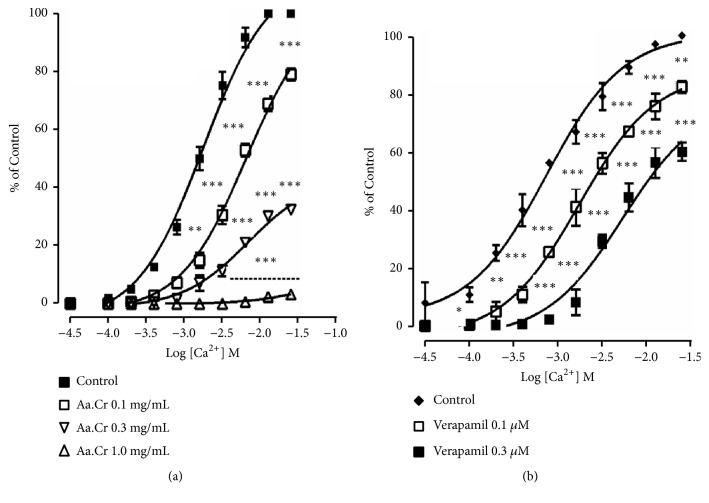
Graph showing concentration response curves (CRCs) of calcium (a) in the absence and presence of Aa.Cr (b) in the absence and presence of verapamil in isolated rabbit jejunum preparation. Curves were constructed in Ca^++^-free but K^+^-rich Tyrode's solution. Data values are presented as mean ± SEM of 6-8 individual experiments and statistically analyzed with the Two-Way ANOVA followed by Dunnett's multiple comparison test.  ^*∗*^ *P*<0.05,  ^*∗∗*^ *P*<0.01,  ^*∗∗∗*^ *P*<0.001 show a comparison of doses with respect to control.

Concentration dependent inhibition of spontaneous contractions and high-K^+^ induced contractions in isolated piece of rabbit jejunum was observed by the application of verapamil-a standard calcium channel antagonist. Similarly, inhibition of spontaneous contractions and high-K^+^ induced contractions in isolated rabbit jejunum was observed after the application of Aa.Cr. Observed respective EC_50_ values of Aa.Cr were 0.98 ± 0.60 mg/mL and 0.40 ± 0.12 mg/ mL. Similar results were observed by Aa.DCM but at lower concentration having respective EC_50_ values 0.45 ± 0.09 mg/mL and 0.22 ± 0.07 mg/mL against spontaneous contractions and high-K^+^ induced contractions in isolated jejunum of rabbit (Figure 1). While Aa.Aq caused spasmogenesis against spontaneously contracting isolated rabbit jejunum, this spasmogenic effect was partially blocked with pretreatment of atropine (1*μ*M) (data not shown), a cholinergic antagonist. The behavior of the jejunum observed after the application of Aa.Cr and Aa.DCM showed similarity to the behavior of calcium channel blockers, i.e., verapamil. To confirm the presence of calcium antagonistic activity in Aa.Cr, concentration response curves (CRCs) of calcium were constructed in the presence and absence of Aa.Cr (0.1-1.0 mg/ mL, n= 4) or verapamil (0.01-0.03 *μ*M, n = 4) in a Ca^++^ free but K^+^-rich Tyrode's solution. The CRCs were shifted towards right with inhibition of maximum contractile effect (Figure 2).

#### 3.2.2. Relaxant Effect on Isolated Rat Trachea

**Figure 3:**
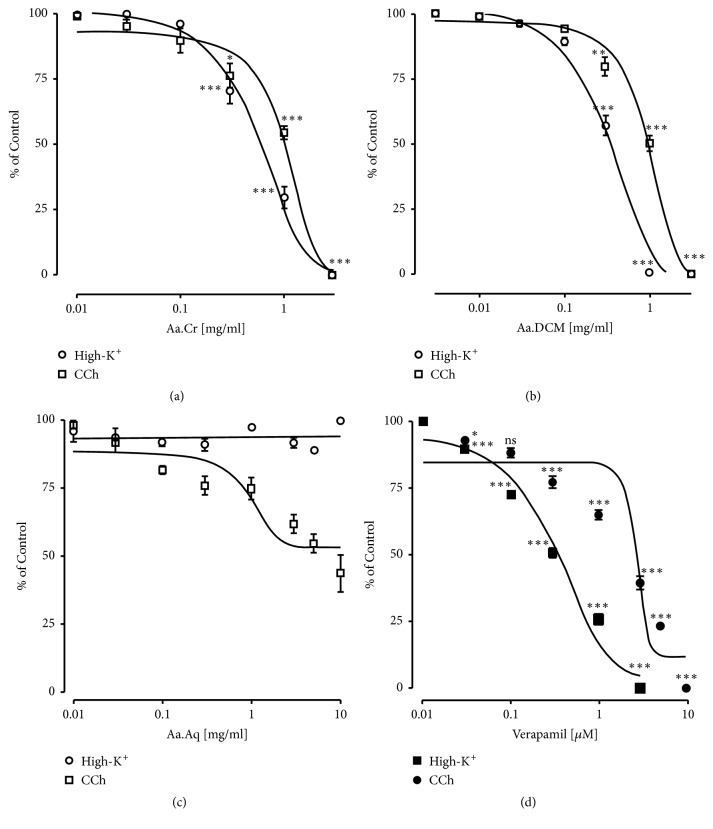
Concentration response curves showing the relaxant effect of (a) Aa.Cr, (b) Aa.DCM, (c) Aa.Aq, and (d) Verapamil against high-K^+^ and carbachol (CCh) induced contractions in isolated rat tracheal tissue. Data values are represented as mean ± SEM of 5-8 individual experiments. Two-Way ANOVA followed by Tukey's post-test was utilized to determine the significant difference among various doses of extract on carbachol and high-K^+^ induced contraction. ^*∗*^ *P*<0.05,  ^*∗∗*^ *P*<0.01,  ^*∗∗∗*^ *P*<0.001 indicate the specified effect compared with the effect of preceding dose.

Tracheal relaxant effect was observed by the application of Aa.Cr to precontracted isolated trachea (Figure 3). EC_50_ value of Aa.Cr against the contractions induced by the application of high-K^+^ was 0.90±0.06 mg/mL and 3.96±0.16 mg/mL against CCh induced contractions. Aa.DCM also showed relaxant effect on high-K^+^ and CCh induced tracheal constriction but at low concentration with EC_50_ values of 0.37±0.08 mg/mL and 1.58±0.06 mg/mL. respectively. Similar response of the tissue was observed by the application of verapamil. While Aa.Aq caused relaxation of high-K^+^ induced contractions in isolated rat trachea, no effect was observed on CCh induced contractions (Figure 3).

#### 3.2.3. Vasodilation Effect

**Figure 4:**
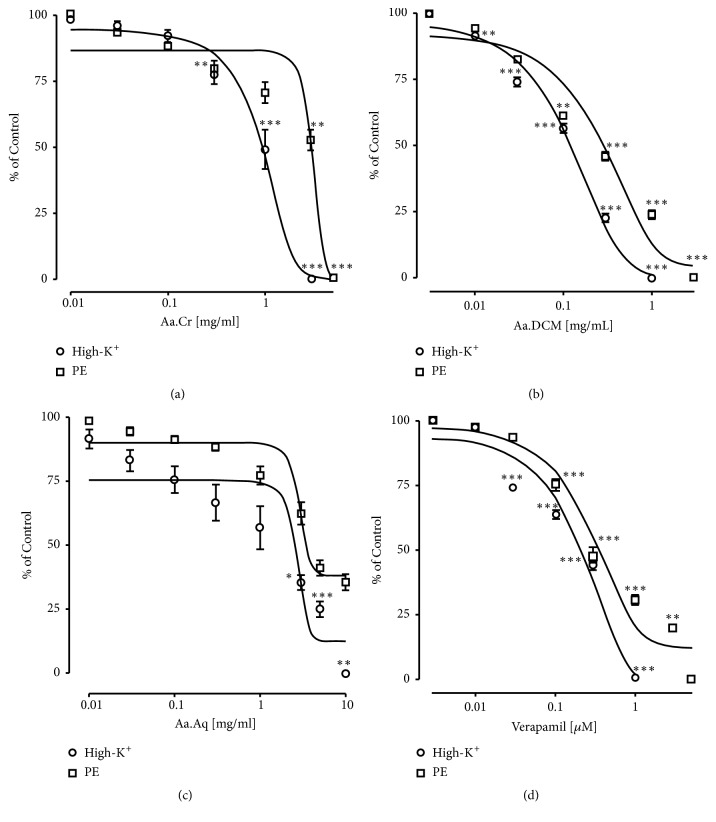
Concentration response curves showing the vasodilation effect of (a) Aa.Cr, (b) Aa.DCM, (c) Aa.Aq, and (d) Verapamil against high-K^+^ and phenylephrine (PE) induced vasoconstriction in isolated rat aorta. Data values are represented as mean ± SEM of 5-8 individual experiments. Two-Way ANOVA followed by Tukey's multiple comparison post-test was utilized to determine the level of significance ( ^*∗*^ *P*<0.05,  ^*∗∗*^ *P*<0.01 ^*∗∗∗*^ *P*<0.001) among various concentrations of extract, when challenged with phenylephrine (PE) and high-K^+^ induced contraction.

To study the vasodilation potential of extract, contractions were induced in isolated rat aortic tissues by the application of high-K+ and PE. When Aa.Cr was applied to these precontracted aortic tissues, concentration dependent vasodilation was observed. EC_50_ value of Aa.Cr against high-K^+^ vasoconstrictions was 1.09±0.07 mg/mL and against phenylephrine (PE) induced vasoconstrictions was 4.76±0.13 mg/mL. But Aa.DCM showed similar results but at lower concentrations with EC_50_ values of 0.13±0.06 mg/mL against high-K^+^ induced vasoconstrictions and 0.29±0.09 mg/mL against PE induced vasoconstrictions. Aa.Aq caused 100% relaxation of high-K^+^ induced vasoconstrictions at the concentration of 10.00 mg/mL with EC_50_ value of 2.42±0.10 mg/mL, but 64.41±2.97% relaxation with EC_50_ value of 3.58 ± 0.11 mg/mL was observed against PE induced constrictions (Figure 4).

#### 3.2.4. Effect on Isolated Atria of Rabbit

**Figure 5:**
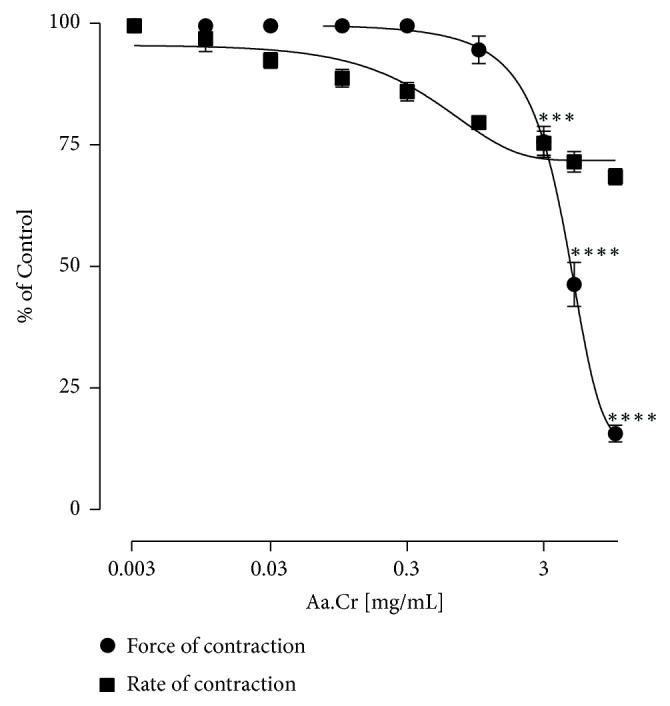
Graph showing the concentration dependent effect of Aa.Cr on isolated rabbit atrium (Data sets are represented as mean ±SEM of 5-6 individual experiments). Two-Way ANOVA followed by Tukey's post-test was utilized to determine the significant difference among various concentrations (^*∗∗∗*^ *P*<0.001).

Rabbit atrium was used to study the effect of test drugs on cardiac rate and force of contractions. When Aa.Cr was applied cumulatively to carefully isolated rabbit atrium, concentration dependent decrease in force of contraction was observed with EC_50_ value of 4.09 mg/mL (3.71-4.40), but effect on rate of contraction was not prominent (Figure 5).

#### 3.2.5. Platelet Aggregation Inhibitory Response

**Figure 6:**
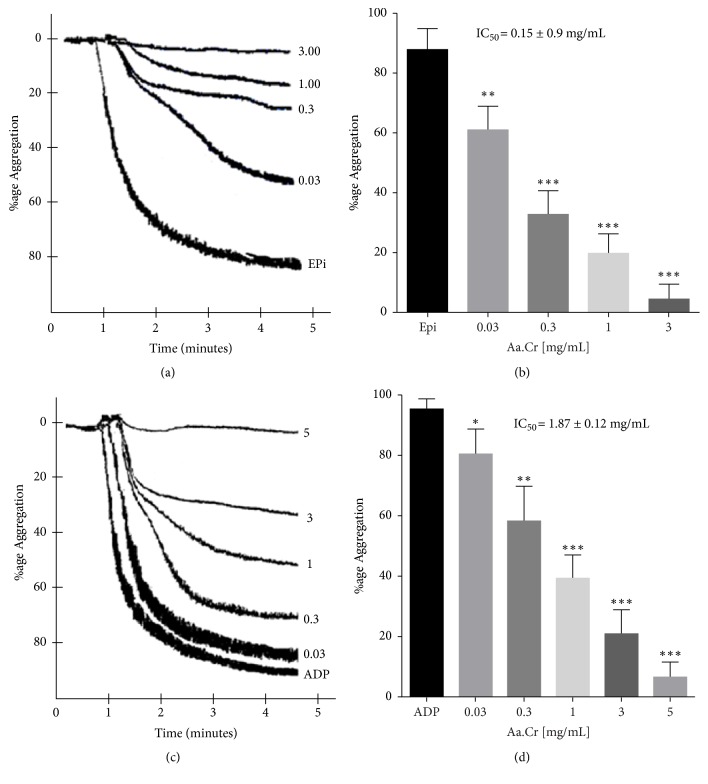
Original tracings (a and c) and Bar graph (b and d) showing the concentration dependent platelet aggregation inhibitory effect of Aa.Cr against epinephrine (EPi) and ADP induced platelet aggregation (data represented as mean ± SEM, n = 5-7 individual experiments). One-Way ANOVA followed by Dunnett's multiple comparison test was used to compare the response of various concentrations with respect to control aggregation by epinephrine (20 *μ*M) and ADP (5 *μ*M). *∗*P<0.05, *∗∗*P<0.01, *∗∗∗*P<0.001 as compared to control values.

Concentration dependent inhibition of platelet aggregation was observed against epinephrine (Epi) and ADP induced aggregations (Figure 6). Respective IC_50_ values of Aa.Cr against EPi and ADP induced aggregation were 0.15 ± 0.9 mg/mL (mean ± SEM, n = 4) and 1.87 ± 0.12 mg/mL (mean ± SEM, n = 5-7).

### 3.3. In Vivo Activities

#### 3.3.1. Antidiarrheal Effect

**Figure 7:**
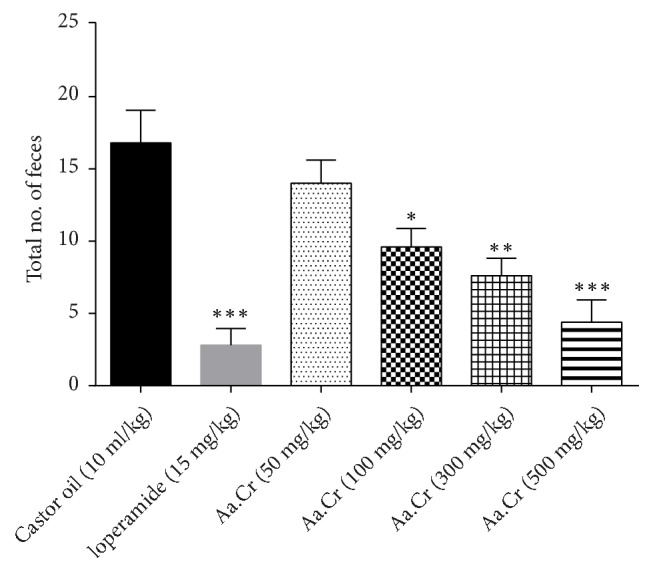
Bar graph indicating antidiarrheal effect of Aa.Cr. Diarrhea was induced by oral administration of castor oil 10 ml/kg. Data sets are presented as mean ± SEM and One-Way ANOVA followed by Dunnett's multiple comparison test was applied for data analysis, *∗P* < 0.01, *∗∗P* < 0.001, *∗∗∗P* < 0.0001 in comparison with control.

Aa.Cr when given orally to the mice which were already treated with castor oil (10 mg/kg) protected them from diarrhea and this protection was dose dependent. Nonsignificant protection was observed in the group treated with Aa.Cr 50 mg/kg while the protection observed at the doses of 100 mg/kg (*P* ≤ 0.01), 300 mg/kg (P ≤ 0.001), and 500 mg/kg (P ≤ 0.001) was significant like verapamil 10mg/kg (P ≤ 0.001) as shown in Figure 7.

#### 3.3.2. Invasive Hypotensive Activity

Normotensive anesthetized rats have been used usually for the study of hypotensive effect of test drugs. When Aa.Cr was given intravenously to preanesthetized rats, dose dependent hypotensive response was evident (Table 1). Significant reduction in blood pressure parameters was observed as compared to normotensive control. Aa.Cr at the dose of 3 mg/kg decreased systolic blood pressure (SBP) to 139.81±3.41 mmHg versus normal SBP 149.00±3.05 mmHg, diastolic blood pressure (DBP) to 101.80±4.18 mmHg versus normal DBP 110.00±5.29mmHg, and mean arterial pressure (MAP) to 114.48±3.45mmHg versus normal MAP 123.45±4.53 mmHg, while Aa.Cr at the dose of 10 mg/kg decreased SBP to 120.25±4.11 mmHg versus normal SBP, DBP to 80.32±5.04 mmHg versus normal DBP, and MAP to 93.63±3.69 mmHg versus normal MAP. Maximum reduction of blood pressure was observed at the dose of 30 mg/kg. Reduction of SBP by the administration of Aa.Cr 30 mg/kg was up to 75.65±3.04 versus normal SBP, DBP was reduced to 48.86±2.45 mmHg versus normal DBP, and reduction of MAP was up to 57.79±2.61 mmHg versus normal MAP.

## 4. Discussions

*Ailanthus altissima* is a member of Simaroubaceae family and is wildly used in traditional medicines and homeopathy for the treatment of various disorders including gastrointestinal, respiratory, cardiovascular, neurological, and peripheral disorders. This study was framed to give the pharmacological proofs for its antidiarrheal, antiasthmatic, hypotensive and platelet aggregation inhibitory effects by using both in vitro and in vivo techniques. Its primary phytochemical investigation showed the presence of alkaloids, saponins, triterpenoids, tannins, glycosides, and flavonoids. Previously reported literature indicates that flavonoids, alkaloids, and tannins have a potential for the relaxation of smooth muscles [34–37].

Free cytosolic calcium has a vital role in regulation of smooth muscle contraction and the entry of this calcium into the cell is through voltage operated calcium channels (L-Type) [38]. Free calcium into the cytoplasm activates ryanodine receptors on sarcoplasmic reticulum and results in the opening of calcium channels present on the membrane of sarcoplasmic reticulum. Calcium rushes into the cytoplasm from these sarcoplasmic stores. This calcium binds with calmodulin and calcium calmodulin complex results. This complex activates an enzyme myosin light chain kinase (MLCK). MLCK activates myosin heads and results in the formation of cross bridges and contraction of the smooth muscles occurs. Contraction of vascular smooth muscles increases peripheral vascular resistance which leads to increase in blood pressure whereas contraction of bronchial smooth muscles results in bronchoconstriction and asthma. Similarly contraction of gastrointestinal smooth muscles leads to diarrhea [25, 39]. Blockage of calcium channels inhibits the influx of calcium into the cytoplasm which results in the relaxation of smooth muscles and this blockage of voltage operated calcium channels is beneficial for the treatment of smooth muscle disorders like asthma, diarrhea, and hypertension [40, 41]. Application of high concentration of potassium (high-K^+^) to smooth muscle tissues results in the opening of voltage operated calcium channels which leads to smooth muscle contraction [42]. Calcium channel blockers have inhibitory role against both spontaneously contracting smooth muscles and smooth muscles preexposed to high-K^+^ but prominent effect against high potassium induced contractions as compared to inhibition of spontaneous contractions [43, 44]. Similarly when Aa.Cr was tested against spontaneous contractions and the contractions induced by the application of high-K^+^ in isolated rabbit jejunum, inhibition of both contractions was observed but inhibitory response was prominent against the contractions induced by high-K^+^ as observed by the verapamil. Similar response was observed by the application of Aa.DCM to the isolated jejunum of rabbit but at lower concentration than Aa.Cr. Blockage of calcium channels was further confirmed when preincubated jejunum was treated with calcium; inhibition of calcium contractile effect was a result with the shifting of concentration response curves of calcium towards right. These in vitro results were confirmed by protection against castor oil induced diarrhea. Calcium channel blockers routinely used for the treatment of gastrointestinal disorders like diarrhea and dysentery and the presence of calcium channel blocking activity in* A. altissima* provide a mechanistic proof for its use in the treatment of diarrhea. Aa.Aq showed spasmogenic effect when applied to isolated jejunum which was blocked with pretreatment of tissue with atropine. These findings showed that spasmolytic and spasmogenic potential is present in* A. altissima* but spasmolytic effect is dominant. This type of behavior (having both agonistic and antagonistic effects) is present in most herbal drugs which is beneficial to overcome the adverse effect of the herbal drugs [45].

*A. altissima* has been used to treat respiratory tract like cold, cough, and asthma [46]. For the validation of its traditional uses its crude extract (Aa.Cr) was applied to tracheal strip isolated from SD rat and showed relaxant effect against high-K^+^ induced and carbachol (CCh) induced tracheal contractions. Potent effect against high-K^+^ induced contractions as compared to the effect against CCh induced contractions was seen like verapamil. High-K^+^ is famous to induce contractions in smooth muscles by opening voltage gated calcium ion channels [47]. Carbachol (CCh) has a reputation to induce contractions in isolated trachea by binding with and stimulating M_3_ receptors. M_3_ receptors are muscarinic receptors which when stimulated result in activation of an enzyme phospholipase C [48]. Phospholipase C converts phosphoinositol 4,5-bisphosphate to inositol 1,4,5-triphosphate (IP_3_) and diacylglycerol (DAG). IP_3_ results in opening of voltage gated calcium channels in cell membrane wile DAG results in the activation of protein kinase C (PKC) [49, 50]. Ultimate result of IP_3_ and DAG formation is the contraction of bronchoconstriction [51]. Aa.Cr caused bronchodilation of both high-K^+^ and CCh induced bronchoconstriction but prominent against high-K^+^ induced bronchoconstriction. Similar behavior was observed by the application of Aa.DCM to isolated tracheal tissues of rat. But Aa.Aq caused bronchodilation of high-K^+^ induced bronchoconstriction, but no effect was observed against CCh induced bronconstriction. These results indicate that bronchodilation is due to the blockage of calcium channels (voltage gated) as observed in the case of rabbit jejunum, thus giving a proof that use of* A. altissima* for the treatment of asthma is due to the blockage of calcium channels. These results also showed smooth muscle relaxant effect of crude effect of* A. altissima* is concentrated in its organic fraction.

*A. altissima* has a reputation to treat cardiovascular problems [13, 52, 53]. Therefore, it was evaluated first on isolated aorta of rat then on rabbit atrium to study its effect on blood vessel tone and cardiac rate and force of contraction. Aa.Cr when applied on phenylephrine and high-K^+^ induced vasoconstrictions showed a concentration dependent vasorelaxant effect. The relaxant effect was more dominant against high-K^+^ induced vasoconstriction indicating that calcium channel blocker effect is dominant mechanism as observed in the case of jejunum and trachea. Similarly, dichloromethane fraction of crude extract (Aa.DCM) also showed relaxation of precontracted aortic tissues. Aa.Aq showed complete relaxation of high-K^+^ induced vasoconstrictions and incomplete relaxation of PE induced vasoconstrictions. Vasorelaxant effect of Aa.DCM was dominant over Aa.Cr and Aa.Aq showing that smooth muscle relaxant effect is concentrated in dichloromethane fraction as compared to aqueous fraction. Relaxation of blood vessels results in decrease in peripheral vascular resistance. When applied to isolated atrium of rabbit, Aa.Cr decreased rate and force of contractions and the results were dependent on concentration. Decrease in cardiac rate and force of contraction may lead to reduction of cardiac output. Blood pressure is directly proportional to peripheral vascular resistance (PVR) and cardiac output (CO). Any drug which increases PVR or CO results in increased BP, but a drug which decreases PVR or CO results in decreased BP [54–58]. As observed above, Aa.Cr possesses vasodilation potential which may cause decrease in PVR and BP. The above cardiovascular results were confirmed in vivo. Intravenous application of Aa.Cr to preanesthetized rats showed concentration dependent fall in blood pressure. These results justify the traditional use of* A. altissima* in cardiovascular disorders like hypertension. In the light of these results, we can say that spasmolytic, bronchodilator, vasorelaxant, and hypotensive activities of* A. altissima* may be due to the blockage of voltage operated calcium channels, but the involvement of other ion channels and receptors cannot be ruled out.

Platelets play an important role in hemostasis maintenance [59] by the aggregation and formation of platelet plug. Uncontrolled aggregation of platelets may lead to progression of various cardiovascular disorders like stroke, atherosclerosis, myocardial infarction, and hypertension [60, 61]. ADP and EPi play an important role in the aggregation of platelets. ADP binds with G-protein coupled receptors called P_2_Y_1_ and P_2_Y_12_. ADP has agonistic effect on these receptors. When ADP binds with its receptors, stimulation of receptors takes place. Stimulation of P_2_Y_1_ results in the release of Ca^++^ via diacylglycerol (DAG) and inositol triphosphate (IP_3_) pathway, but activation of P_2_Y_12_ receptors results in inactivation of adenylyl cyclase enzyme which leads to inhibition of release of cAMP. Released calcium activates platelets and events of aggregation start up [62–64]. Epinephrine causes aggregation of platelets by stimulation of *α*_2_-receptors [65–67]. Concentration dependent inhibition of aggregation was seen when ADP and EPi were applied to PRP incubated with Aa.Cr, but the effect against EPi was more potent. Therefore, it may be said that Aa.Cr caused inhibition of platelet aggregation by antagonizing P_2_Y_1_, P_2_Y_12,_ and *α*_2_-receptors. As it has been observed in our isolated tissue experiments, Aa.Cr possessed calcium channel blocking activity, so the involvement of calcium channel potential cannot be ignored.

It has been reported that phytochemical constituents like alkaloids, flavonoids, and tannins present in herbal drugs possess smooth muscle relaxant effects [36, 68, 69]. We have also observed that Aa.Cr contains alkaloids, tannins, and flavonoids. So it can be said that the observed smooth muscle relaxant effects may be due to the presence of these phytochemicals.

## 5. Conclusion

In short, it can be concluded that* Ailanthus altissima* may possess calcium antagonistic compounds which could mediate the relaxation of jejunum, tracheal and aortic tissue in ex vivo experiments. These compounds may belong to battery of alkaloids, tannins, and saponins, but the role of other phytochemical constituents and involvement of other ion channels and receptors cannot be ruled out. Inhibition of platelet aggregation may be due to antagonistic effect on P_2_Y_1_, P_2_Y_12_, and *α*_2_-receptors and therefore downstream inhibition of calcium channel pathways would be a potential target of Aa.Cr. However, this is the preliminary part of experiments and further investigation deems necessary to explore the mechanistic pathways linked with the identification and isolation of active compound (s) from* Ailanthus altissima.*

## Figures and Tables

**Table 1:** Hypotensive effect of crude extract of *Ailanthus altissima *(Aa.Cr) in mmHg on various blood pressure parameters in normotensive anesthetized rats.

Group	SBP	DBP	MAP
Control	149.0 ± 3.05	110.0 ± 5.29	123.45 ± 4.53
Verapamil (1 mg/kg)	84.33 ± 3.48	63.67 ± 2.96	70.56 ± 3.12
Aa.Cr (3 mg/kg)	139.81±3.41	101.80±4.18	114.48±3.45
Aa.Cr (10 mg/kg)	120.25±4.11^*∗∗*^	80.32±5.04^*∗*^	93.63±3.69^*∗∗*^
Aa.Cr (30 mg/kg)	75.65±3.04^*∗∗∗*^	48.86±2.45^*∗∗∗*^	57.79±2.61^*∗∗∗*^

Data are expressed as mean ±SEM (*n* = 5 individual experiments). Student *t*-test was utilized to analyze data, ^*∗*^ *P*<0.05, ^*∗∗*^ *P*<0.01, ^*∗∗∗*^ *P*<0.001.

## Data Availability

The data used to support the findings of this study are available from the corresponding author upon request.

## List of Abbreviations

### Abbreviations

Aa.Aq: Aqueous fraction of Aa.Cr
Aa.Cr: Crude extract of* Ailanthus altissima*
Aa.DCM: Dichloromethane fraction of Aa.Cr
ADP: Adenosine diphosphate
CCh: Carbachol
CRC: Concentration response curves
DAG: Diacylglycerol
DBP: Diastolic blood pressure
EPi: Epinephrine
High-K^+^: High concentration of potassium (K-80 mM)
IP3: Inositol triphosphate
MAP: Mean arterial pressure
PE: Phenylephrine
PPP: Platelet poor plasma
PRP: Platelet rich plasma
SBP: Systolic blood pressure.
